# Polymorphisms in interleukin 17A gene and coal workers’ pneumoconiosis risk in a Chinese population

**DOI:** 10.1186/s12890-015-0076-1

**Published:** 2015-07-30

**Authors:** Ruhui Han, Xiaoming Ji, Baiqun Wu, Ting Wang, Lei Han, Jingjin Yang, Baoli Zhu, Chunhui Ni

**Affiliations:** Department of Occupational Medicine and Environmental Health, School of Public Health, Nanjing Medical University, Nanjing, 210029 China; Institute of Occupational Disease Prevention, Jiangsu Provincial Center for Disease Control and Prevention, Nanjing, China

**Keywords:** Genetics, *IL-17A*, Polymorphisms, Coal workers’ pneumoconiosis

## Abstract

**Background:**

The interleukin 17A (*IL-17A*) which is located on chromosome 6p and has been linked to chronic inflammation, is an important candidate gene conferring coal workers’ pneumoconiosis (CWP). The purpose of this study was to investigate the genetic association between single nucleotide polymorphisms (SNPs) of *IL-17A* and CWP in a Chinese population.

**Methods:**

We conducted a case–control study to investigate the role of four common SNPs in the *IL-17A* gene, and evaluated the relationship between these four SNPs and dust-exposure year, tobacco smoking and stages of CWP. A total of 1391 subjects was enrolled in this study, including 694 subjects in control group and 697 in case group. TaqMan based qRT-PCRs were taken to genotype rs2275913, rs3748067, rs4711998, and rs8193036 within the *IL-17A* gene. Luciferase assays were used to determine the effects of rs8193036 C > T alleles on the expression of *IL-17A.*

**Results:**

Unconditional logistic regression analysis showed that the genotypes of rs3748067 AA (adjusted OR = 0.43, 95 % CI = 0.23–0.83) and rs8193036 TT (adjusted OR = 0.59, 95 % CI = 0.40–0.86) were associated with a decreased risk of CWP, particularly among subgroups of smokers (adjusted OR =0.34, 95 % CI = 0.13–0.86 for rs3748076; adjusted OR = 0.41, 95 % CI = 0.23–0.71 for 8193036) and CWP cases with stage I (adjusted OR = 0.45, 95 % CI = 0.21–0.98 for rs3748076; adjusted OR = 0.46, 95 % CI = 0.28–0.74 for 8193036). Furthermore, the polymorphism of rs3748067 significantly reduced the CWP risk among cases with over 27 years of dust exposure (adjusted OR = 0.42, 95 % CI = 0.18–0.97). The luciferase assays in two cell lines showed that the rs8193036 C > T substitution could reduce the expression of *IL-17A*, which was consistent with the findings of our association study.

**Conclusions:**

The rs3748067 G > A and rs8193036 C > T polymorphisms decrease CWP risk. These findings could be helpful in identifying individuals at decreased risk for CWP and further studies are warranted to validate them.

## Background

Coal workers’ pneumoconiosis (CWP), common occurred in underground coal miners, is one of the most prevalent occupational diseases in China. CWP is characterized by chronic lung inflammation and formation of fibrotic nodular lesions that results from the inhalation of airborne coal mining dust which usually contains free crystalline silica [1, 2]. CWP is a kind of progressive and irreversible fibrotic lung diseases without any effective therapy. It was reported that cytokines, such as transforming growth factor-β1 (TGF-β1), interleukin (IL)-1β, IL-6 and IL-13, were produced by alveolar macrophages to promote lung inflammation after silica inhalation. Persistent inflammation was converted into lung fibrosis eventually [3, 4]. Many factors are related to the prevalence of CWP, including the long time exposure to high concentrations of respirable crystalline silica [5, 6], poor personal protection and individual susceptibility [7].

Interleukin-17A (*IL-17A*) is a cytokine with strong pro-inflammatory effect and has been reported to be elevated in fibrotic disorders [8, 9]. *IL-17A* is important for host defense against extracellular pathogens and it can lead to drastic inflammatory responses by recruiting neutrophils and other cytokines [10]. Thus, *IL-17A* may play a crucial role in the inflammation of silicosis [11]. *IL-17A* is produced by a subset of CD4+ T-helper (Th) cells, Th17 cells, which are distinct from the classic Th1 and Th2 cells. Therefore, *IL-17A* plays an important role in both innate and adaptive immunities [12, 13]. However, *IL-17A* is secreted not only by Th17 cells, but by γδ T-cells and natural killer T-cells as well [14, 15]. Previous studies about *IL-17A* in CWP were rare whereas the effect of *IL-17A* in lung inflammation and fibrosis was definite. Increased levels of *IL-17A* have been demonstrated in bronchoalveolar lavage (BAL) of patients with IPF and lung tissues exposed to BLM and IL-1β [16]. Neutralization of *IL-17A* could delay the progression of silica-induced lung inflammation and fibrosis in C57BL/6 mice [17, 18]. A huge number of studies have identified the association between the *IL-17A* polymorphisms and risk for human disorders, such as pediatric asthma [19], multiple sclerosis [20], gastric cancer [21], and dilated cardiomyopathy [22]. In the present study, we attempted to clarify the association between *IL-17A* polymorphisms and CWP risk in a Chinese population.

## Materials and methods

### Study population

Our study population consisted of 697 CWP patients and 694 controls. They were recruited from the coal mines of Xuzhou Mining Business Group Co., Ltd. between January 2006 and December 2010, as described previously [23]. Briefly, all participants were genetically unrelated Chinese Han males and were underground coal miners who spent their entire working career within the above mentioned company. The subjects with clinical evidence of autoimmunity diseases, had received immunosuppressive or immunostimulatory therapy, or were subjected to radiotherapy were excluded. High kilovolt chest X-ray and physical examinations were performed based on the China National Diagnostic Criteria for Pneumoconiosis (GBZ 70–2002), which are the same as that of the 1980 International Labour Organization (ILO) in the judgment of opacity profusion,to reconfirm the diagnoses [24] . According to the size, profusion, and distribution of opacities, all patients were classified into stage I, stage II or stage III. The chest X-rays were assessed by at least two independent physicians. The questionnaire for each participant was conducted by the face-to-face interview using a double-blind method. This epidemiological questionnaire focused on age, respiratory symptoms, occupational histories, and smoking habits and some others. Blood sample of 5 ml was obtained from each participant, and was used for routine lab tests. The control subjects were miners matched with the CWP cases for age, dust exposure period, and job types from the same company in order to make the dust exposure histories between cases and controls were comparable. This study protocol was specifically approved by the Institutional Review Board of Nanjing Medical University and all subjects gave their written informed consent before participating in the study.

### Genotyping

Conventional phenol-chloroform method was used to extract the genomic DNA from peripheral blood lymphocytes. Genotyping was performed using the TaqMan method with the ABI 7900HT Real Time PCR system according to the manufacturer’s instructions (Applied Biosystem, Foster city, CA, USA) in a blinded fashion, which means the people conducted the genotyping experiment was unaware of the workers’ personal details or case status. The sequences of primer and probe for each SNP are available on request. Genomic DNA (50 ng) was used for each reaction, and amplification was performed under the following conditions: 50 °C for 2 min and 95 °C for 10 min followed by 45 cycles of 95 °C for 15 sec and 60 °C for 1 min. Negative controls were included in each plate to ensure accuracy of the genotyping. 10 % of the samples were randomly selected for confirmation, and the results were 100 % concordant.

### Cell culture

Human lung adenocarcinoma A549 cells and human bronchial epithelial (HBE) cells were purchased from the Shanghai Institute of Biochemistry and Cell Biology, Chinese Academy of Science (Shanghai, China). Cells were cultured in Dulbecco modified Eagle medium supplemented with 100U/ml penicillin, 100 μg/ml streptomycin, and 10 % fetal bovine serum. The cells were grown at 37 °C in the presence of 5 % carbon dioxide in a humidified incubator.

### Construction of luciferase reporter plasmids

We constructed 2 luciferase reporter plasmids to explore whether rs8193036C > T polymorphism had an effect on *IL-17A* gene expression in vitro. The constructs of a 792-bp DNA fragment corresponding to the upstream region of the transcription start site of *IL-17A*, which were amplified from individual homozygous templates and were cloned into the pGL3-basic luciferase vector (Promega, Madision, WI). The vectors were then sequenced to confirm that there were no nucleotide errors.

### Transient transfections and luciferase assays

A549 and HBE cells were seeded in 24-well plates, and each well was transfected with 2.4 μg of the vector DNA containing either C or T allele of rs8193036 and 0.08 μg of pRL-SV40 which contains the Renilla luciferase gene by Lipofectamine 2000 (Invitrogen, Carlsbad, CA), according to the manufacture’s instruction. Cells were collected 48 h after transfection, and luciferase activity was measured with a dual Luciferase reporter assay system (Promega) and was normalized against the activity of the Renilla luciferase gene. Independent triplicate experiments were performed for each plasmid.

### Statistical analyses

Differences in the distributions of demographic characteristics, selected variables, and frequencies of genotypes of *IL-17A* polymorphisms between the CWP cases and controls were evaluated by using the Student’s *t*-test or *χ*^2^-test. The Hardy-Weinberg equilibrium (HWE) was tested using a goodness-of-fit *χ*^2^-test. The associations between genotypes and CWP were estimated by computing odds ratios (ORs) and their 95 % confidence intervals (CIs) from unconditional logistic regression analysis with the adjustment for possible confounders.

The statistical power was calculated by using the PS software (http://biostat.mc.vanderbilt.edu/twiki/bin/view/Main/PowerSampleSize). For the stratified analysis, the age and dust-exposure cutoff was according to the median of age and dust-exposure years of the participants. All statistical tests were two-sided at a significance level of 0.05 and were analyzed by the SAS software (version 9.1; SAS Institute, Inc.,Cary, NC).

## Results

Four *IL-17A* SNPs were genotyped in 697 CWP patients and 693 controls. The information about frequency distributions of the selected characteristics was summarized in Table 1. There were no significant difference between the cases and controls in the distribution of age (*P* = 0.103), exposure years (*P* = 0.105), and job types (*P* = 0.534). Although the smoking status of CWP was similar to the controls (*P* = 0.250), the smoking amount (pack-years) in CWP cases was significantly less than that of controls (*P* < 0.001). The frequency distributions and means of the selected characteristics were matched adequately between cases and controls. Furthermore, of the 697 CWP cases, the pneumoconiosis stages from I to III were 415 (59.5 %), 219 (31.4 %) and 63 (9.0 %).

**Table 1:** Demographic and selected variables among the CWP cases and controls

Variables	CWP (*n* = 697)		Controls (*n* = 694)		*P*
	Number	Percent	Number	Percent	
Age, year (mean ± SD)	68.0 ± 11.1		67.1 ± 8.4		0.103
Exposure years (mean ± SD)	26.6 ± 9.0		27.3 ± 7.8		0.105
Smoking status					
Never	340	48.8	360	51.9	0.250
Ever	357	51.2	334	48.1	
Former	163	23.4	91	13.1	
Current	194	27.8	243	35.0	
Pack-years smoked					<0.001
0	340	49.2	360	52.6	
0-20	223	32.0	132	19.0	
>20	134	19.2	202	29.1	
Work type					0.534
Tunnel and coal mining	663	95.1	652	94.0	
Transport	16	2.3	17	2.5	
Others	18	2.6	25	3.6	
Stage					
I	415	59.5			
II	219	31.4			
III	63	9.0			

The primary information and allele frequencies observed were listed in Table 2. All genotyped distributions of control subjects were consistent with those expected from the Hardy-Weinberg equilibrium. The minor allele frequencies (MAF) of these four polymorphisms were consistent with that reported in the HapMap database (http://www.hapmap.org).

**Table 2:** Primary information of genotyped SNPs in IL-17(IL-17 polymorphisms)

rs no.	Location	Base change	MAF		HWE^a^
			Case	Control	
rs2275913	5’UTR	G > A	0.440	0.435	0.393
rs3748067	3’UTR	G > A	0.160	0.189	0.135
rs4711998	5’UTR	A > G	0.284	0.285	0.852
rs8193036	5’UTR	C > T	0.289	0.333	0.493

^a^HWE *P* value in the control group

Logistic regression analysis was performed to assess the effect of each SNP on CWP risk (adjusting for age, exposure years, job type, and pack-years of smoking). Parameters for the association of SNPs with CWP were shown in Table 3. The analysis revealed that two SNPs (rs3748067, rs8193036) of *IL-17A* were associated with the risk of CWP significantly. It was revealed that the variant allele decreased the susceptibility to CWP under co-dominant (OR = 0.43, 95 % CI = 0.22–0.81, *P* = 0.010 for AA versus GG for rs3748067; OR = 0.60, 95 % CI = 0.41–0.87, *P* = 0.007 for TT versus CC for rs8193036), recessive (OR = 0.44, 95 % CI = 0.23–0.83, *P* = 0.012 for rs3748067; OR = 0.64, 95 % CI = 0.44–0.91, *P* = 0.014 for rs8193036), and additive (OR = 0.82, 95 % CI = 0.67–0.99, *P* = 0.012 for rs3748067; OR = 0.81, 95 % CI = 0.69–0.95, *P* = 0.011 for rs8193036) models. These associations remained significant after adjusting for age, exposure years, and pack-years smoking. Carrying the A allele of rs3748067 was associated with decreased risk (adjusted OR = 0.81, 95 % CI = 0.66–0.99). Similarly, subjects with the *IL-17A* rs8193036 T allele showed a lower decreased risk relative to the C allele, with adjusted OR = 0.80, 95 % CI = 0.68–0.95, respectively. However, no association was found between rs2275913 G > A, rs4711098 A > G polymorphism and CWP occurrence.

**Table 3:** Distributions of genotypes of *IL-17* and their associations with risk of CWP

Variables	CWP cases		Controls		*P* ^a^	OR (95 % CI)	*P* ^b^	OR (95 % CI)^b^
	Number	Percent	Number	Percent				
rs2275913	*n* =692		*n* = 687					
GG	221	31.9	225	32.8		1.00		1.00
AG	333	48.1	326	47.5	0.749	1.04 (0.82-1.32)	0.665	1.05 (0.83-1.34)
AA	138	19.9	136	19.8	0.832	1.03 (0.76-1.40)	0.734	1.05 (0.78-1.43)
G allele	775	56.0	776	56.5		1.00		1.00
A allele	609	44.0	598	43.5	0.800	1.02 (0.88-1.19)	0.695	1.03 (0.89-1.20)
ADD					0.802	1.02 (0.88-1.18)	0.699	1.03 (0.88-1.20)
DOM					0.746	1.03 (0.83-1.30)	0.647	1.05 (0.84-1.32)
REC					0.946	1.01 (0.77-1.32)	0.880	1.02 (0.78-1.33)
rs3748067	*n* = 693		*n* = 690					
GG	486	70.1	460	66.7		1.00		1.00
GA	193	27.8	199	28.8	0.476	0.92 (0.73-1.16)	0.408	0.90 (0.71-1.15)
AA	14	2.0	31	4.5	0.010	0.43 (0.22-0.81)	0.011	0.43 (0.23-0.83)
G allele	1165	84.1	1119	81.1		1.00		1.00
A allele	221	15.9	261	18.9	0.040	0.81 (0.67-0.99)	0.035	0.81 (0.66-0.99)
ADD					0.041	0.82 (0.67-0.99)	0.036	0.81 (0.67-0.99)
DOM					0.166	0.85 (0.68-1.07)	0.139	0.84 (0.67-1.06)
REC					0.012	0.44 (0.23-0.83)	0.014	0.44 (0.23-0.85)
rs4711998	*n* = 693		*n* = 689					
AA	350	50.5	353	51.2		1.00		1.00
GA	293	42.3	279	40.5	0.610	1.06 (0.85-1.31)	0.558	1.06 (0.86-1.33)
GG	50	7.2	57	8.3	0.556	0.88 (0.59-1.33)	0.628	0.90 (0.60-1.36)
A allele	993	71.6	985	71.5		1.00		1.00
G allele	393	28.4	393	28.5	0.924	0.99 (0.84-1.17)	0.984	1.00 (0.85-1.18)
ADD					0.923	0.99 (0.84-1.17)	0.984	1.00 (0.85-1.18)
DOM					0.787	1.03 (0.83-1.27)	0.711	1.04 (0.84-1.29)
REC					0.462	0.86 (0.58-1.28)	0.518	0.88 (0.59-1.31)
rs8193036	*n* = 693		*n* = 690					
CC	347	50.1	311	45.1		1.00		1.00
TC	292	42.1	298	43.2	0.252	0.88 (0.70-1.10)	0.233	0.87 (0.70-1.09)
TT	54	7.8	81	11.7	0.007	0.60 (0.41-0.87)	0.006	0.59 (0.40-0.86)
C allele	986	71.1	920	66.7		1.00		
T allele	400	28.9	460	33.3	0.011	0.81 (0.69-0.95)	0.009	0.80 (0.68-0.95)
ADD					0.011	0.81(0.69-0.95)	0.009	0.81 (0.69-0.95)
DOM					0.062	0.82 (0.66-1.01)	0.053	0.81 (0.66-1.00)
REC					0.014	0.64 (0.44-0.91)	0.011	0.63 (0.43-0.90)

*ADD* wild homozygote versus heterozygote versus mutational homozygote, *DOM* wild homozygote versus heterozygote and mutational homozygote, *REC* wild homozygote and heterozygote versus mutational homozygote

^a^Two-sided *χ* ^2^ test

^b^Adjusted for age, exposure years, jobtype, and pack-years of smoking in logistic regression model

We further investigated the effect of exposure years, smoking status and stage of CWP on the association between *IL-17A* gene polymorphisms and CWP risk. As shown in Table 4, the association between rs3748067 and CWP risk remained significant among subjects who had longer than 27 years of exposure (OR = 0.42, 95 % CI = 0.18–0.97, *P* = 0.041) under a recessive model. However, the variants rs8193036 significantly decreased CWP risk of individuals who had less than 27 years of exposure (OR = 0.46, 95 % CI = 0.25–0.85, *P* = 0.014) under a recessive model (Table 5). In addition, the variants rs3748067 and rs8193036 both significantly decreased CWP risk of smokers (OR = 0.34, 95 % CI = 0.13-0.86, *P* = 0.022 for rs3748067; OR = 0.41, 95 % CI = 0.23-0.71, *P* = 0.002 for rs8193036) under a recessive model. Additionally, significant associations were observed between the genotypes and patients with stage I (OR = 0.45, 95 % CI = 0.21–0.98, *P* =0.045 for rs3748067; OR = 0.46, 95 % CI = 0.28–0.75, *P* =0.001 for rs8193036) under a recessive model. Furthermore, case-control logistic regression analysis was conducted to investigate the interaction between SNP and smoking status. Nevertheless, no variants was interacted with smoking status significantly (OR =0.71, 95 % CI = 0.20-2.56, *P* =0.596 for rs3748067; OR =0.60, 95 % CI = 0.29-1.24, *P* = 0.163 for rs8193036; Table 6).

**Table 4:** Stratification analyses between the genotypes of rs3748067 and CWP risk

Variables	Cases/controls	Genotypes (cases/controls)				*P* ^a^	OR (95 % CI)^a^
		GG/GA		AA			
		*n*	%	*n*	%		
Total	693/690	679/659	98.0/95.5	14/31	2.0/4.5	0.014	0.44 (0.23-0.85)
Age							
<68	276/405	270/388	97.8/95.8	6/17	2.2/4.2	0.040	0.33 (0.11-0.95)
≥68	417/285	409/271	96.9/95.1	8/14	1.9/4.9	0.041	0.68 (0.16-0.96)
Exposure years							
<27	270/267	264/255	97.8/95.5	6/12	2.2/4.5	0.210	0.52 (0.19-1.44)
≥27	423/423	415/404	95.5/98.1	8/19	1.9/4.5	0.041	0.42 (0.18-0.97)
Smoking status							
never	340/359	333/345	98.0/96.1	7/14	2.1/3.9	0.228	0.56 (0.22-1.43)
ever	353/331	346/214	am	7/17	2.0/5.1	0.022	0.34 (0.13-0.86)
Stage							
I	415/690	406/659	97.8/95.5	9/31	2.2/4.5	0.045	0.45 (0.21-0.98)
II	217/690	212/659	97.7/95.5	5/31	2.3/4.5	0.199	0.52 (0.19-1.41)
III	61/690	61/659	100/95.5	0/31	0/4.5		

^a^Adjusted for age, exposure years, jobtype, and pack-years of smoking in logistic regression model

**Table 5:** Stratification analyses between the genotypes of rs8193036 and CWP risk

Variables	Cases/controls	Genotypes (cases/controls)				*P* ^a^	OR (95 % CI)^a^
		CC/TC		TT			
		Number	Percent	Number	Percent		
Total	693/690	639/609	92.2/88.3	54/81	7.8/11.7	0.011	0.63 (0.43-0.90)
Age							
<68	276/405	258/354	93.5/87.4	18/51	6.5/12.6	0.025	0.50 (0.27-0.92)
≥68	417/285	381/255	91.4/89.5	36/30	8.63/10.5	0.335	0.78 (0.47-1.30)
Exposure years							
<27	270/267	253/233	93.7/82.3	17/34	6.3/12.7	0.014	0.46 (0.25-0.85)
≥27	423/423	386/376	91.3/88.9	37/47	8.8/11.1	0.227	0.75 (0.48-1.19)
Smoking status							
never	340/359	309/319	90.9/88.9	31/40	9.1/11.1	0.448	0.82 (0.50-1.36)
ever	353/331	330/290	93.5/87.6	23/41	6.5/12.4	0.002	0.41 (0.23-0.71)
Stage							
I	415/690	391/609	94.2/88.3	24/81	5.8/11.7	0.001	0.46 (0.28-0.74)
II	217/690	196/609	90.3/88.3	21/81	9.7/11.7	0.437	0.81 (0.48-1.37)
III	61/690	52/609	85.3/88.3	9/81	14.8/11.7	0.445	1.36 (0.62-2.96)

^a^Adjusted for age, exposure years, and pack-years of smoking in logistic regression model

**Table 6:** Interaction between *IL-17A* SNPs (rs3748067 and rs8193036) and smoking on CWP risk: case–control analysis

Interaction markers	β	OR_i_ (95 % CI)^a^	*P* ^a^
rs3748067–smoking	−0.348	0.71 (0.20 – 2.56)	0.596
rs8193036–smoking	−0.521	0.60 (0.29 – 1.24)	0.163

^a^Adjusted for age, exposure years, and job type in logistic regression model

**Fig. 1:**
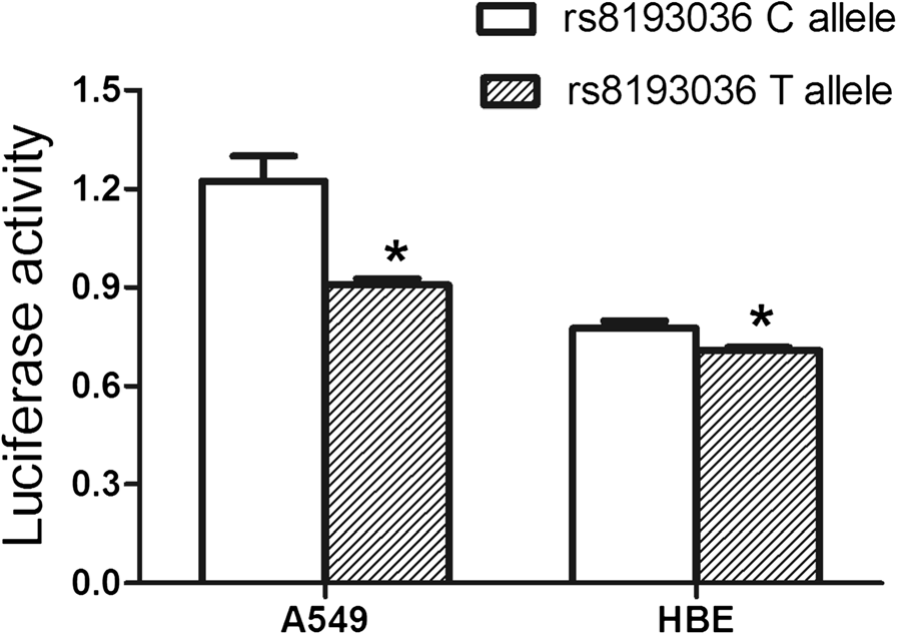
Two constructs were transiently transfected into A549 and HBE cells, respectively. Luciferase activity of each construct was normalized against internal control of Renilla luciferase. Data indicated mean values with SD from 3 independent experiments. ^*^ *P* < 0.05 compared with construct counterpart

In order to evaluate whether the *IL-17A* rs8193036 C > T polymorphism is associated with the transcriptional activity of *IL-17A*, pGL3-basic vectors with either rs8193036 C or rs8193036 T allele were constructed. The above vectors were transfected into A549 or HBE cells, respectively. As shown in Fig. 1, the vectors with rs8193036 T allele reduced the relative luciferase activities significantly, compared with the rs8193036 C allele in both of the cell lines (*P* < 0.05). These results suggested that the rs8193036 T allele in the 5’UTR region was associated with a decreased transcriptional activity of *IL-17A*.

## Discussion

This study was investigated to explore the probable relationship between *IL-17A* genetic variants and the susceptibility to CWP. We found a remarkable association between two SNPs (rs3748067 and rs8193036) of *IL-17A* gene and the resistance to the disease, and the associations were more evident in smokers. Similarly, subjects carrying the rs3748067 AA and rs8193036 TT genotypes had a moderately decreased risk of CWP patients with stage I. Luciferase assays demonstrated that *IL-17A* promoters with rs8193036 T allele decreased the transcriptional activity of *IL-17A*, compare with rs8193036 C allele, which was consistent with the findings in our relevant study.

CWP is a serious occupational disease which is common occurred in underground coal miners. There is no effective treatment for CWP currently. The incidence and progression of CWP are determined by both dust exposure levels [5] and silica content in the dust [24]. However, only few people exposed to coal dust or silica developed CWP ultimately. Thus, characterization and identification of genes involved in the genetic predisposition or progression have an important role in clinical settings for the treatment of CWP [25].

IL-17 is a cytokine released from Th17 and other IL-17 producing cells, IL-17 cytokine family consists of six members including *IL-17A* (*IL-17*), *IL-17B*, *IL-17C*, *IL-17D*, *IL-17E* (also known as *IL-25*) and *IL-17 F*. Many cells are *IL-17A*-responsive, such as epithelial cells, endothelial cells, fibroblasts, macrophages, and dendritic cells. Furthermore, Th17 cell and other cells which as producers of *IL-17A* are also sensitive to the cytokine [26]. *IL-17A* plays a crucial role in the development and progression of both acute and chronic inflammation-induced pulmonary fibrosis. It may modulate the inflammatory response and synthesis of collagen [17, 27]. In addition, the elevated expression of *IL-17A* has been reported in animal models of fibrotic disease and in human fibrotic tissues, including lungs [28], livers [29], skin [9] and others.

*IL-17A* participates in the pathogenesis of fibrotic disorders maybe through three pathways. First, *IL-17A* can regulate lung inflammation through an IL-1β dependent mechanism [9]. A previous study has described that innate *IL-1β*-*IL-23*-*IL-17A* axis in the establishment of early pulmonary inflammation with direct consequences on late evolution to fibrosis after lung injury. Their data suggest that lung injury promotes IL-1β production which increases IL-23 expression and in turn can stimulate innate IL-17 expression [30]. Mark et al. [16] identified that genetic deletion of *IL-17A* significantly attenuated lung inflammation and fibrosis induced by BLM treatment. Second, *IL-17A* can stimulate collagen synthesis from fibroblasts, directly and/or indirectly, via fibroblast production of cytokines such as TGF-β1 and connective growth factor (CTGF) [17]. *IL-17A* increased the synthesis and secretion of collagen and promoted the epithelial-mesenchymal transition in alveolar epithelial cells in a TGF-β1 dependent manner [17]. Taiji et al. [31] reported that the *IL-17A* signaling pathway took an antifibrogenic effect in scleroderma fibroblasts as intrinsic activation of TGF-β1 inhibits *IL-17A* signaling by the down-regulation of the receptor and contributed to the excess collagen accumulation and tissue fibrosis via miR-129-5p. Third, *IL-17A* could upregulate the expression of tissue inhibitor of metalloproteinase-1 (TIMP-1), and inhibited extracellular matrix (ECM) degradation by matrix metalloproteinases (MMPs) effectively [32].

To our knowledge, this is the first evaluation of the association between functional SNPs in *IL-17A* and CWP susceptibility in a Chinese population. Based on the above-mentioned studies, it seems that *IL-17A* may have a vital role in the pathogenesis and development of CWP. Investigation of *IL-17A* gene polymorphisms in patients with CWP and controls showed that the inheritance rate of rs3748067 AA genotype and rs8193036 TT genotype were more frequent in the controls than in the patients, thus they may be resistance factors for CWP. Furthermore, stratification analyses were applied, and each of these two SNPs (rs3748067 and rs8193036) significantly decreased CWP risk of individuals who has ever smoked.

Functional analyses of the 2 related *IL-17A* polymorphisms showed that rs8193036 C > T influenced lower transcription of *IL-17A* in vitro. Rs3748067 was a locus of expression for interferon regulatory factor 4 (IRF4), a transcription factor that has been innumerable linked to the transient inflammation and progressive fibrosis [33]. Rs8193036 may be located on a transcription factor binding site (TFBS), affecting transcription activity and it may predispose individuals to fibrogenesis. These findings provide new insights into the role of *IL-17A* in the pathogenesis and development of CWP.

Several limitations of this present study should be considered. First, we matched the dust exposure levels by job titles and exposure years between case and control, and therefore, the selection bias could not be ruled out and the subjects could not fully represent the general populations of coal miners in China. Second, although our study suggested that rs3748067 and rs8193036 variations of *IL-17A* gene were associated with the risk of CWP, more biological background data and functional studies are needed to explain the results. Third, the sample size of this study is relatively moderate, which may reduce the statistical power to find the other difference between groups. Therefore, further large sample size studies with more diverse ethnic populations are required to replicate our results.

## Conclusion

Taken together, the present study first indicates that two functional *IL-17A* SNPs (rs3748067 and rs8193036) are associated with decreased risk of CWP in a Chinese population, especially among the subgroup of smokers and patients with stage I. Further prospective study and strict case–control study are warranted to confirm our findings. Actually, the control of dust exposure in the workplace is the only way to totally eliminate pneumoconiosis.

## Abbreviations

*IL-17A*: Interleukin 17A
CWP: Coal workers’ pneumoconiosis
SNPs: Single nucleotide polymorphisms
COPD: Chronic obstructive pulmonary disease
BAL: Bronchoalveolar lavage
IPF: Idiopathic pulmonary fibrosis
HWE: Hardy-Weinberg equilibrium
ORs: Odds ratios
CIs: Confidence intervals
MAF: Minor allele frequency
BLM: Bleomycin
TGF-β1: Transforming growth factor-β1
CTGF: connective growth factor
TIMP-1: Tissue inhibitor of metalloproteinase-1
ECM: Extracellular matrix degradation
MMPs: Matrix metalloproteinases
IRF4: Regulatory factor 4
TFBS: Transcription factor binding site
